# Increased Frequency of Circulating Follicular Helper T Cells in Children with Hand, Foot, and Mouth Disease Caused by Enterovirus 71 Infection

**DOI:** 10.1155/2014/651872

**Published:** 2014-06-11

**Authors:** Jianping Wu, David Cui, Xianzhi Yang, Jianzhou Lou, Jie Lin, Xianfei Ye, Zhimei Qin, Li Huang, Dejian Zhao, Zhaoxia Huo, Guoliang Xie, Shufa Zheng, Fei Yu, Liwei Lu, Yu Chen

**Affiliations:** ^1^Department of Clinical Laboratory, The First Affiliated Hospital, School of Medicine, Zhejiang University, 79 Qingchun Road, Hangzhou 310003, China; ^2^Department of Clinical Laboratory, Center of Community Health Service of Qingbo Street, Hangzhou 310002, China; ^3^Department of Pathology and Center of Infection and Immunology, The University of Hong Kong, Hong Kong

## Abstract

Enterovirus 71 (EV71) is a major causative agent of hand, foot, and mouth disease (HFMD) in children. The role of T follicular helper (TFH) cells in EV71-infected children remains unclear in regulating humoral immunity. The frequency of circulating ICOS^high^/PD-1^high^CXCR5^+^CD4^+^ TFH cells in the children with mild and severe EV71 infection and healthy controls (HC) was detected by flow cytometry, respectively. IL-21 and IL-6 mRNA expression and their serum levels, Bcl-6 mRNA expression, and specific neutralizing antibodies against EV71 (NAb-EV71) were measured. In the acute stage of patients with EV71 infection, increased frequencies of circulating TFH cells with ICOS^high^ and PD-1^high^ expression in the mild and severe patients were observed, and the positive correlations among the frequencies of circulating TFH cells and the serum levels of IL-21, IL-6, and NAb-EV71 titres were detected, respectively. Moreover, the expressions of IL-6 and IL-21 mRNA in PBMCs from patients were also significantly higher than those of HC. However, further analysis did not reveal any significant differences between mild and severe patients. These data indicate a role of TFH cells and associated cytokines in modulating the humoral response during the pathogenesis of EV71 infection.

## 1. Introduction

Enterovirus 71 (EV71) is a positive-stranded RNA genome and belongs to a member of the species A,* Enterovirus* genus, Picornaviridae family [1]. EV71 is one of the major causative agents of hand, foot, and mouth disease (HFMD) in young children, which has caused a series of outbreaks of HFMD throughout the world, with particular prevalence in Asian-Pacific region [2–6]. Most HFMD cases are mild, and self-limited disease is caused mainly by EV71 and Coxsackievirus A16 (CA16), but HFMD cases caused by EV71 have resulted in severe neurological complications, pulmonary edema, and fetal death; EV71 has become a serious public health concern [5–8]. Currently, there is no safe and effective vaccine available for prevention and control of this disease. There is also no approved antiviral drug for treatment of EV71 infection. Recent studies suggest that neutralizing antibodies (NAb) against EV71 (NAb-EV71) are crucial for the protection from EV71 infection in animals and young children [4–6].

In an adaptive immune response, Th1 and Th2 subsets of CD4^+^ helper T cells are considered to play a pivotal role in helping B cells to class-switch Ig isotypes via secreting special cytokines, such as IFN-*γ* and IL-4 and Th1 and Th2 cytokines, respectively [9]. Recently, T follicular helper (TFH) cells have been clearly described as a specialized subset of CD4^+^ T cells, which distinguish from Th1, Th2, Th17, and Treg and localize to B cell follicles of germinal center (GC), where they can regulate the humoral immune responses [10–14]. Typical features of TFH cells are the expression of chemokine (C-X-C motif) receptor 5 (CXCR5), inducible costimulator (ICOS), programmed death-1 (PD-1), interleukin- (IL-) 21, and B-cell lymphoma 6 (BCL-6) [14–16]. Additionally, TFH cells also express other surface molecules including CD40 ligand (CD40L), OX40, IL-21 receptor (IL-21R), and IL-6 receptor (IL-6R) [14]. TFH cells can migrate to C-X-C motif chemokine 13 (CXCL13) expressed on B cells through CXCR5, which is carried out in GC, where TFH cells and relative cytokines regulate the development of B-cells responses as well as Ig isotype switching and the production of optimal antibodies [17–20]. Studies indicated that ICOS and PD-1 of the CD28 family members are closely correlated with the functions of TFH cells; some cytokines such as IL-6, IL-12, IL-21, and IL-23 can induce IL-21 secretion in human naïve CD4^+^ T cells, but only IL-12 induces the sustained expression of CXCR5 and ICOS on these activated naïve CD4^+^ T cells, which promotes TFH cell development by upregulation of BCL-6 expression [20–25]. Bcl-6 is an essential transcriptional factor that directly affects TFH cells differentiation and represses transcriptional regulators of other Th cells [17].

Recent studies have shown that circulating TFH cells were dysregulated in patients with lymphoma, autoimmune diseases, and several infectious diseases [25–30]. Increasing evidence indicates that Th1, Th2, Th17, and Treg subsets and B cells are critically involved in the pathogenesis of EV71 infection [31–35]. However, the role of circulating TFH cells is not yet characterized in regulating humoral immune response in EV71-infected children. In this study, we reported that the frequency of circulating TFH cells and levels of NAb-EV71, IL-21, and IL-6 mRNA expression were significantly increased in patients at the acute stage of EV71 infection, compared to HC. Additionally, the frequency of circulating CXCR5^+^CD4^+^ TFH cells with ICOS^high^ and PD-1^high^ was positively correlated with levels of IL-21, IL-6, and NAb-EV71. These data indicated that the TFH cell might play a crucial role in the pathogenesis of HFMD at acute stage of EV71 infection.

## 2. Materials and Methods

### 2.1. Patients

A total of 60 children below ten years with EV71 infections acquired during outbreaks between April and September 2013 in the First Affiliated Hospital of College of Medicine, Zhejiang University. The diagnoses were established depending on clinical features and laboratory criteria. EV71-infected HFMD was defined by EV71-positive throat swabs, stools, rectal swabs, vesicular swabs, or cerebrospinal fluid (CSF) samples which were detected using specific EV71 primers and probe from the enterovirus 71 nucleic acid detection kit (Da An Gene Co., Ltd., Guangzhou, China). Mild case was characterized by typical manifestations with or without fever, which usually recovered spontaneously within one week; severe case was defined as a febrile exanthematous disease with neurological signs of encephalitis, meningitis, and/or cardiopulmonary complications, as previously described [3]. Clinical samples and data were collected from the EV71-infected HFMD children who had not been treated at the time the samples were collected in the first diagnose in the hospital, and children with bacterial infections were excluded.

43 healthy children with age and gender matched were included in this study as controls from health checks requiring blood puncture on parents' request or physicians' advice to test for HBV infection status, liver function, and blood routine examination at our hospital. According to the Declaration of Helsinki (1964), informed consents were obtained from the parents or guardians of the children, and the medical ethical committee of our hospital approved this study. Main clinical data of these children are shown in Table 1.

### 2.2. Cell Isolation

Peripheral blood samples were obtained from the healthy children and the patients in the acute stage of EV71 infection, and peripheral blood mononuclear cells (PBMCs) were isolated by density-gradient centrifugation using Ficoll-Hypaque solution.

### 2.3. Flow Cytometric Analysis

Human PBMCs were washed and stained with PerCP/cy5.5-anti-CXCR5 and APC-anti-CD4, PE-anti-CD278 (ICOS), and FITC-anti-CD279 (PD-1) (Biolegend, San Diego, CA, USA). Isotype-matched Ab controls were used in all procedures. All the staining was performed according to manufacturer's protocol. The frequency of circulating TFH cells was determined by a FACSCalibur flow cytometry (Beckton Dickinson, BD Bioscience, USA) and FlowJo software version 7.6.5 (TreeStar, San Carlos, CA).

### 2.4. RNA Isolation and Real-Time PCR

For detection of* IL-6, IL-21,* and* BCL-6* mRNA expression, total RNA from PBMCs was extracted with Trizol (Invitrogen, USA). cDNA was synthesized by reverse transcription reagent kits (Takara, Dalian, China) according to the manufacturer's instruction. Real-time PCR was performed in triplicate using Takara SYBR super mix (Takara, Dalian, China) by ABI 7500 analysis system (Applied Biosystems, CA, USA). Amplified conditions were as follows: 5 min at 95°C for denaturation, then 40 cycles of 95°C for 10 sec and 60°C for 40 sec, and collecting for fluorescence at 60°C. Primer sequences were as follows:* IL-6* sense: 5′-GGCCTTCCCTACTTCACAAG-3′, antisense: 5′-ATTTCCACGATTTCCCAGAG-3′;* IL-21* sense: 5′-AGATCCAGTCCTGGCAACATG-3′, antisense: 5′-GGCAGAAATTCAGGGACCAAG-3′; and* BCL-6* sense: 5′-TTGTGAGCCGTGAGCAGTTT-3′, antisense: 5′-TGTCTTGGGGCATCAGCAT-3′. Each gene was normalized using **β*-actin* gene with the following primers: sense, 5′-TGGAATCCTGTGGCATCCATGAAAC-3′; antisense, 5′-TAAAACGCAGCTCAGTAACAGTCCG-3′. Data were analyzed by ABI 7500 Software (Applied Biosystems, CA, USA).

### 2.5. Enzyme-Linked Immunosorbent Assay (ELISA)

Serum samples were stored at −80°C. The levels of the sera IL-6 and IL-21 in individual children and healthy controls were measured by enzyme-linked immunosorbent assay (ELISA) using LEGEND MAX Human IL-6 ELISA Kit with Pre-coated Plates and LEGEND MAX Human IL-21 ELISA Kit with Pre-coated PlatesELISA kits (Biolegend, San Diego, CA, USA) according to the manufacturer's instructions. All samples were run in triplicate.

### 2.6. Neutralizing Antibody (NAb) against EV71 Assay (NAb-EV71)

Neutralizing antibodies (NAb) against EV71 (Zhejiang/DTID/ZJU-74) in sera was detected by a microneutralization test in 96-well plates, as described previously [3]. Briefly, the sera inactivated at 56°C for 30 minutes were serially diluted from 1 : 8 to 1 : 1,024 in DMEM containing 2% fetal bovine serum. Serum dilution (25 *μ*L) and 25 *μ*L viral cultures with 100 fifty percent tissue culture infective dose (TCID_50_) were mixed and incubated at 37°C for 2 hours. The serum-virus mixtures were then added into 100 *μ*L of human rhabdomyosarcoma (RD) cell suspension (2 × 105 cells/mL), incubated at 37°C for 3 days in 5% CO_2_, and monitored for characteristic cytopathic effect (CPE). The neutralizing titer was defined as the reciprocal of the dilution of the serum that neutralized ≥50% of the infected cells. If the serum neutralization titer was ≥1 : 8, the specific neutralizing antibody (NAb) against EV71 was considered to be positive. An antibody-negative serum and uninfected cells were defined as controls.

### 2.7. Statistical Analysis

One-way ANOVA analysis was performed to confirm whether there was an overall statistically significant change among the groups. Student's *t*-test was performed as appropriate. Correlations between variables were determined using Spearman's correlation coefficient. Data were analyzed using GraphPad Prism 5 software (GraphPad Software, Inc., San Diego, CA).

## 3. Results

### 3.1. Increased Frequency of Circulating CXCR5^+^CD4^+^ TFH Cells in Children with EV71 Infection

To identify the viral etiology of HFMD from each child in our recruited population, 60 children with EV71-mediated HFMD were identified by one-step real-time RT-PCR assay for at least one of clinical samples from throat swabs, stools, rectal swabs, vesicular swabs, or CSF. 31 cases were considered to be severe HFMD with CNS involvement, and 29 cases were mild according to clinical symptoms (Table 1). We found that severe patients occurred below 3 years; the number of peripheral blood WBC and the concentration of CRP were notably different among the severe, mild, and HC groups.

**Figure 1:**
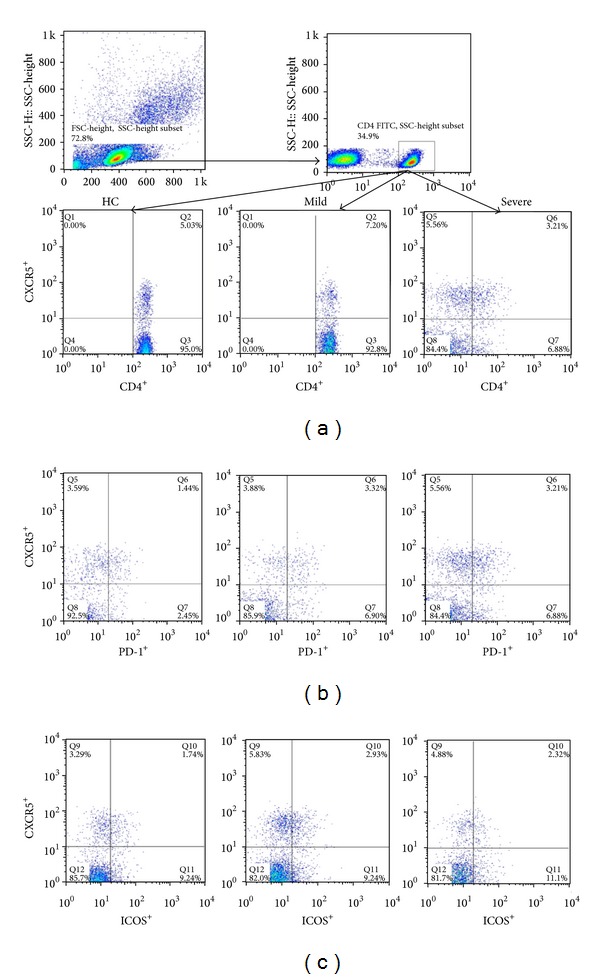
Detection of circulating CXCR5^+^CD4^+^ TFH cells with ICOS^high^ and PD-1^high^ expression in peripheral blood of EV71-infected patients and HC. Peripheral blood mononuclear cells (PBMCs) from patients with EV71 infection and HC were stained with labeled antibodies and analyzed by flow cytometry as described in Section 2. The cells were gated initially on living lymphocytes (upper left) and then on CD4^+^ T cells (upper right). Representative plots of CXCR5^+^CD4^+^ TFH cells in the total CD4^+^ T cells (a); PD-1^high^CXCR5^+^CD4^+^ TFH cells (b); and COS^high^CXCR5^+^CD4^+^ TFH cells (c).

**Figure 2:**
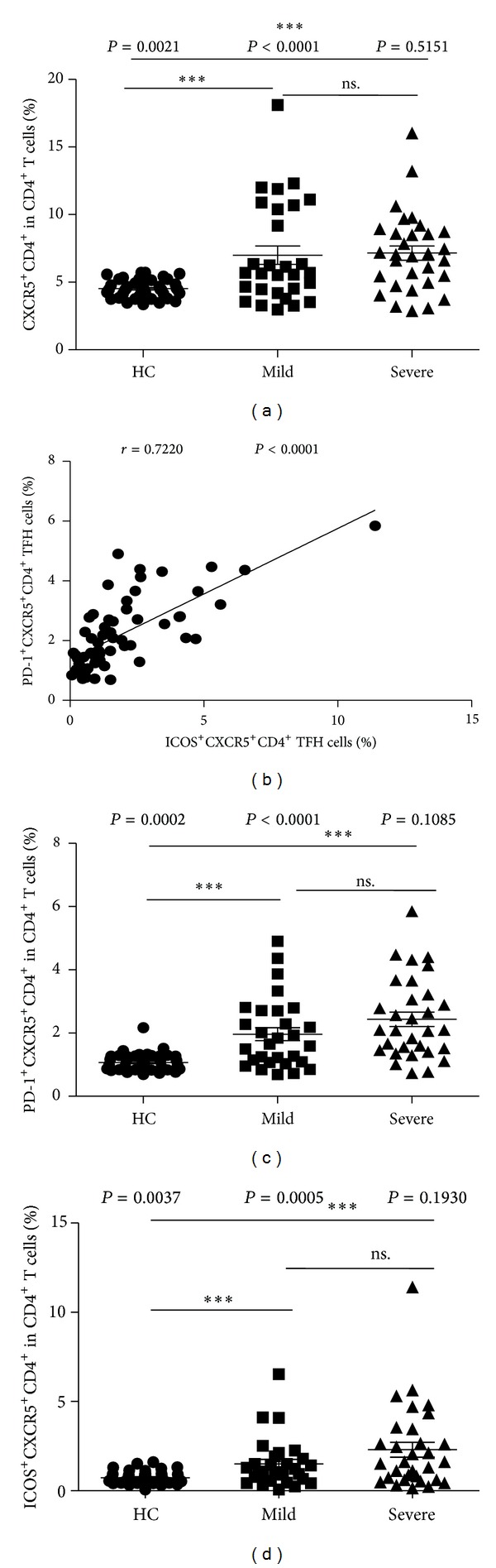
Increased percentages of circulating CXCR5^+^CD4^+^ TFH cells with ICOS^high^ and PD-1^high^ expression in peripheral blood of EV71-infected patients. The percentages of CXCR5^+^CD4^+^ TFH cells in the total CD4^+^ T cells (a); the correlation between the percentages of PD-1^+^CXCR5^+^CD4^+^ TFH cells and ICOS^+^CXCR5^+^CD4^+^ TFH cells from EV71-infected patients (*n* = 60) (b); PD-1^+^CXCR5^+^CD4^+^ TFH cells (c) and ICOS^+^CXCR5^+^CD4^+^ TFH cells (d). Data shown were the mean ± SD (standard deviation, SD). The horizontal lines show the median. *P < 0.05; **P < 0.01; ***P < 0.001; ns.: no significant difference.

To explore the potential role of circulating TFH cells in EV71-mediated HFMD children, the frequency of circulating CXCR5^+^CD4^+^ TFH cells and the percentages of ICOS^high^ or PD-1^high^CXCR5^+^CD4^+^ TFH cells were analyzed using flow cytometry (Figures 1(a)–1(c)). The frequencies of circulating CXCR5^+^CD4^+^ TFH cells in CD4^+^ T cells in both mild (7.00 ± 3.69) and severe (7.15 ± 2.93) HFMD patients were significantly higher than those in HC (4.52 ± 0.65) (Figure 2(a)). Moreover, the frequencies of ICOS^high^ CXCR5^+^CD4^+^ TFH cells (HC: 0.73 ± 0.33; mild: 1.50 ± 1.38; severe: 2.30 ± 2.33) and PD-1^high^CXCR5^+^CD4^+^ TFH cells (HC: 1.07 ± 0.28; mild: 1.96 ± 1.11; severe: 2.44 ± 1.27) in CD4^+^ T cells were similar in both mild and severe cases; they were notably higher than those of HC (Figures 2(c)-2(d)), respectively. However, the percentages of circulating CXCR5^+^CD4^+^ TFH cells, ICOS^high^, or PD-1^high^CXCR5^+^CD4^+^ TFH cells were not significantly different between mild and severe HFMD patients (Figures 2(c)-2(d)). In addition, a strongly positive correlation was observed between ICOS^high^CXCR5^+^CD4^+^ TFH and PD-1^high^CXCR5^+^CD4^+^ TFH cells (*r* = 0.6762; *P* < 0.0001) in EV71-infected patients (Figure 2(b)).

### 3.2. High Levels of Neutralizing Antibodies against EV71 (NAb-EV71) with Increased Circulating TFH Cells in Children with Mild and Severe Infections

**Figure 3:**
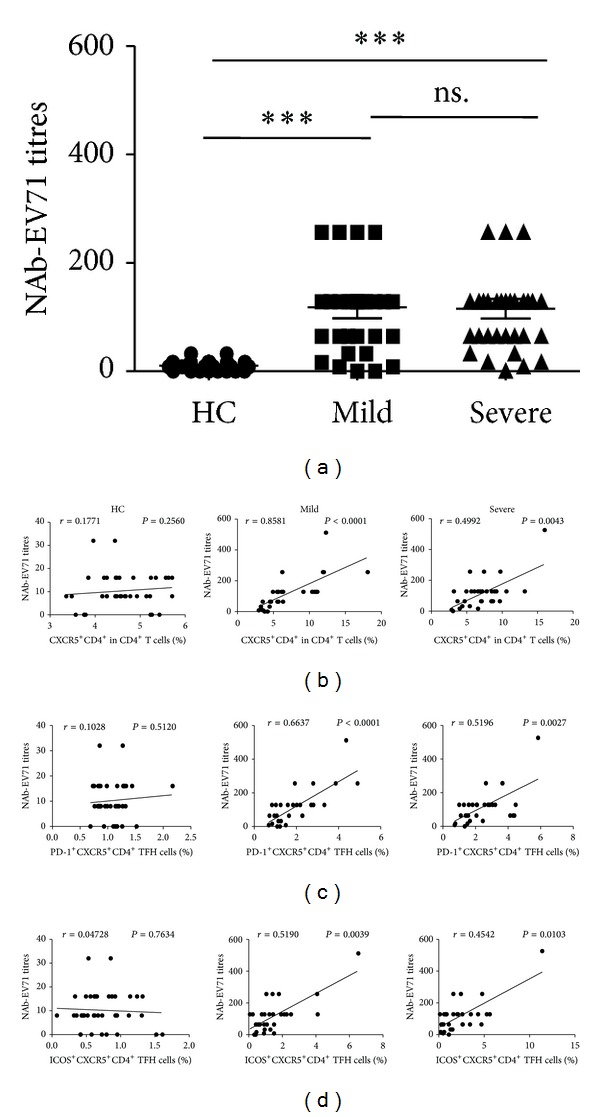
Correlation of specific NAb-EV71 titres and circulating CXCR5^+^CD4^+^ TFH cells with PD-1^high^ and ICOS^high^ expression in EV71-infected patients. (a) Titres of specific NAb-EV71 in sera from HC and EV71-infected patients including mild and severe cases. Relationship between the titres of specific NAb-EV71 and the frequency of circulating CXCR5^+^CD4^+^ TFH cells (b), PD-1^+^CXCR5^+^CD4^+^ TFH cells (c), and ICOS^+^CXCR5^+^CD4^+^ TFH cells (d). Data shown were the mean ± SD. The horizontal lines show the median. *P < 0.05; **P < 0.01; ***P < 0.001; ns.: no significant difference.

The increased frequencies of circulating TFH cells in human autoimmune thyroid disease (AITD) including Grave's disease (GD) and Hashimoto's thyroiditis (HT), rheumatoid arthritis (RA), and systemic lupus erythematosus (SLE) were significantly correlated with the levels of autoantibodies and disease activity [13, 14, 27–30]. Next, we sought to examine whether the frequency of circulating TFH cells was associated with the levels of specific NAb-EV71 in the mild and severe patients with EV71 infection. It was found that the levels of specific NAb-EV71 in both mild and severe EV71-patients were significantly higher than those of HC, but no significant difference was observed between mild and severe patients (HC: 10.23 ± 7.47; mild: 118.07 ± 108.11; severe: 115.29 ± 101.59) (Figure 3(a)). The frequencies of circulating CXCR5^+^CD4^+^ TFH cells, ICOS^high^, or PD-1^high^CXCR5^+^CD4^+^ TFH cells were all positively significantly associated with the levels of specific NAb-EV71 in the mild and severe patients, but not with HC, respectively (Figures 3(b)–3(d)).

### 3.3. Serum Levels of Cytokines Related to Circulating TFH Cells in Children with Mild and Severe Infections

**Figure 4:**
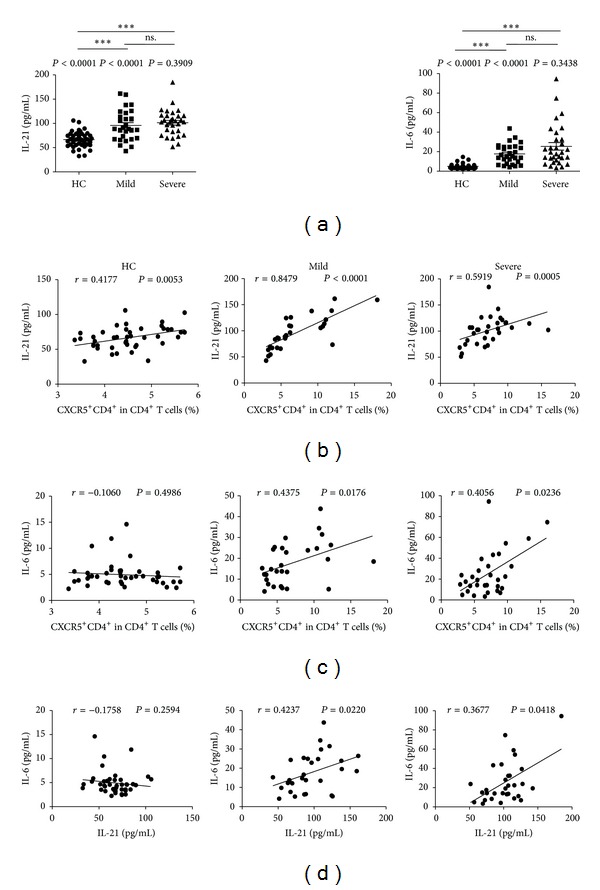
Correlation of cytokines levels and circulating CXCR5^+^CD4^+^ TFH cells in EV71-infected patients including mild and severe cases. (a) Cytokines levels of IL-21 and IL-6 in sera from HC and EV71-infected patients. (b) Relationship between the IL-21 levels and the frequency of circulating CXCR5^+^CD4^+^ TFH cells. (c) Relationship between the IL-6 levels and the frequency of circulating CXCR5^+^CD4^+^ TFH cells. (d) Relationship between the IL-6 levels and IL-21 levels. Data shown were the mean ± SD. The horizontal lines show the median. *P < 0.05; **P < 0.01; ***P < 0.001; ns.: no significant difference.

Recent studies show that cytokines, both interleukin-21 (IL-21) and IL-6, had critical roles in differentiation and function of follicular helper CD4^+^ T cells [14, 21, 30]. The levels of cytokines, both IL-21 (HC: 66.65 ± 15.88; mild: 92.34 ± 33.26; severe: 101.46 ± 26.82) and IL-6 (HC: 4.94 ± 2.43; mild: 17.68 ± 10.03; severe: 25.45 ± 21.52) in sera from the patients with EV-71 infection, were notably higher than those of HC, although no significant difference was found in both mild and severe children, respectively (Figure 4(a)). The levels of serum IL-21 were strongly positively correlated with the frequencies of TFH cells among HC, mild, and severe patients (Figure 4(b)). Interestingly, a significant positive correlation between IL-6 levels and CXCR5^+^CD4^+^ TFH cells frequencies was observed between mild and severe patients, respectively, although it was not found in HC (Figure 4(c)). Interestingly, IL-6 level showed a significant positive correlation with IL-21 level in mild and severe-HFMD patients, respectively (Figure 4(d)).

### 3.4. Correlation between Sera IL-21 and IL-6 and Specific NAb-EV71 in Children with Mild and Severe Infections

**Figure 5:**
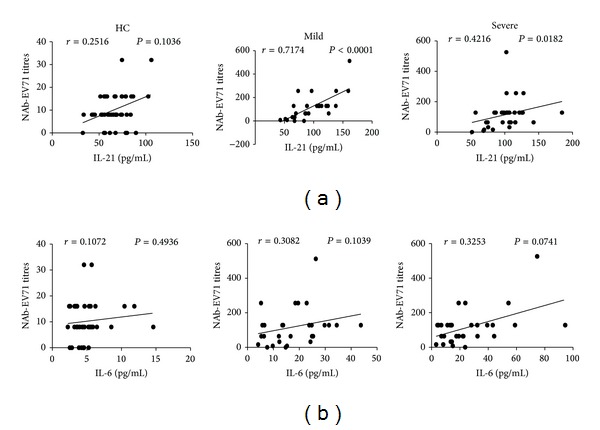
Correlation of specific NAb-EV71 titres and cytokines levels from HC and EV71-infected patients including mild and severe cases. (a) Relationship between the titres of specific NAb-EV71 and the serum levels of IL-21. (b) Relationship between the titres of specific NAb-EV71 and the serum levels of IL-6.

Recent studies demonstrated that the levels of both IL-21 and IL-6 cytokines were significantly associated with antibody expression [14, 21]. In this study, we found that the concentrations of serum IL-21 were positively correlated with that of specific NAb-EV71 in mild or severe patients, although this correlation was not observed in HC (Figure 5(a)). However, no significant association was noted between serum IL-6 levels and specific NAb-EV71 concentrations in any of the three groups tested, respectively (Figure 5(b)).

### 3.5. Expression of Bcl-6, IL-21, and IL-6 mRNA in Children with Mild and Severe Infections

**Figure 6:**
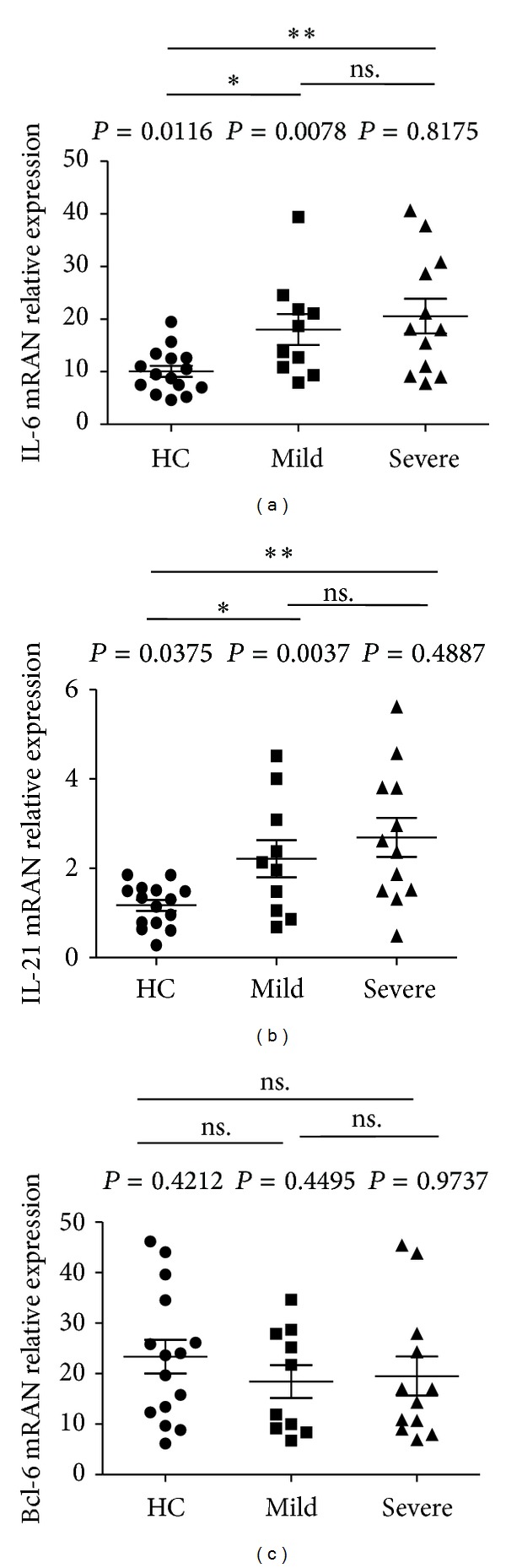
Expression of IL-6, IL-21, and Bcl-6 mRNA in human PBMCs. Human PBMCs from 10 mild and 12 severe patients with EV71 infection, and 15 HC were detected by real-time PCR assay as described in Section 2. (a) The levels of IL-6 mRNA in human PBMCs. (b) The levels of IL-21 mRNA in human PBMCs. (c) The levels of Bcl-6 mRNA in human PBMCs; data shown were the mean ± SD. The horizontal lines show the median. *P < 0.05; **P < 0.01; ***P < 0.001; ns.: no significant difference.

Previous studies indicated that Bcl-6 was a pivotal transcription factor for CD4^+^ T cell differentiation into TFH cells [14, 18, 26]. Both IL-21 and IL-6 share cooperative functions in TFH differentiation and B cell immunity [21]. Here, we assessed the expression of Bcl-6, IL-21, and IL-6 mRNA in HC (*n* = 15), mild (*n* = 10), and severe (*n* = 12) EV71-patients. The IL-6 mRNA expression in severe patients or mild patients was significantly higher than that in HC, but no significant difference of IL-6 mRNA expression was observed between mild and severe patients groups (HC: 10.08 ± 4.16; mild: 18.02 ± 9.41; severe: 20.55 ± 11.38) (Figure 6(a)). Similarly, the IL-21 mRNA expressions in both mild and severe patients were significantly higher than those in HC, but no significant difference of IL-21 mRNA was found between mild patients and severe patients (HC: 1.17 ± 0.48; mild: 2.21 ± 1.31; severe: 2.69 ± 1.51) (Figure 6(b)). Moreover, there were no significant differences for Bcl-6 mRNA expression among these groups tested (HC: 23.34 ± 12.92; mild: 18.45 ± 10.28; severe: 19.54 ± 13.32) (Figure 6(c)).

## 4. Discussion

There are more than one million HFMD cases and 200 deaths each year since 2008 in China [36]. EV71 is one of the common causative factors of HFMD in young children less than 5 years of age [3, 5]. Although mild diseases are the predominant clinical features of EV71 infection, severe diseases with neurological involvement and the fetal cases of HFMD are closely associated with EV71 infection identified by PCR and/or virus isolation [6–8]. In our study, compared to mild patients, the age of severe patients was mainly observed below 3 years (severe versus mild cases, *P* = 0.0016), and evaluated evidences with WBC numbers (severe versus mild cases, *P* < 0.0001; mild cases versus HC, *P* = 0.0035) and serum CRP levels (severe versus mild cases, *P* < 0.0001; mild cases versus HC, *P* < 0.0001) suggested that inappropriate inflammation may be elicited in the progression of HFMD in the acute phase of EV71 infection, which was consistent with previously reported data [7, 31, 32].

Previous studies have demonstrated that both humoral and cellular immune responses, such as antibodies, B and T cells including Th1/Th2 and Th17, and other T cell lineages and related cytokines, play a critical role in protection from EV71 infection in animal models [4, 34, 35]. In humans, EV71 infection induced imbalance of T/B cells and their related cytokines and even caused a systemic dysregulation of the immune system [31–33]. Recent studies show that follicular helper T cells (TFH), a new T cell subpopulation, play a pivotal role in regulating immune responses and have been associated with many diseases [13, 20, 28]. Simpson et al. reported an increased frequency of circulating CXCR5^+^CD4^+^ TFH cells in patients with systemic lupus erythematosus (SLE) and Sjogren's syndrome [30]. Feng et al. demonstrated that the frequency of circulating TFH cells was significantly increased in patients with HBV, and these circulating TFH cells participated in the HBV-related immune responses [28]. However, the potential role of circulating TFH cells in patients with EV71 infection is not yet known. In the present work, we firstly examined the frequency of circulating TFH cells in mild and severe patients with EV71 infection and healthy controls. The results demonstrated that the frequency of circulating CXCR5^+^CD4^+^ TFH cells in mild and severe patients in the acute stage of EV71 infection was significantly higher than that of HC (severe or mild cases versus HC, *P* < 0.05), respectively. However, severe patients did not differ significantly from mild patients in the percentage of peripheral blood CXCR5^+^CD4^+^ TFH cells (severe versus mild cases, *P* = 0.5151). These findings clearly indicated that TFH cells played an important role in the EV71-induced immune responses.

Simpson et al. detected that the high frequency of circulating TFH cells with high expression of ICOS and/or PD-1 molecules was associated with the severity of end-organ involvement in patients with systemic lupus erythematosus (SLE) and Sjogren's syndrome [30]. Zhu et al. demonstrated the increased frequency of TFH cells with highly expressed ICOS and/or PD-1 in patients with autoimmune thyroid disease and rheumatoid arthritis, respectively, and both ICOS and PD-1 molecules are closely positively correlated with the function of TFH cells [13]. We detected an enhanced frequency of PD-1^high^ and ICOS^high^CXCR5^+^CD4^+^ TFH cells in the mild and severe EV71-induced patients, compared to HC (severe or mild cases versus HC, *P* < 0.05). In addition, a closely positive correlation was observed between the frequencies of these two subpopulations of PD-1^high^ and ICOS^high^CXCR5^+^CD4^+^ T cells. These data supported the definition of circulating TFH cells. However, the percentage of circulating TFH cells with ICOS^high^ and PD-1^high^ expression revealed no significant difference between the mild and severe patients (severe versus mild cases, PD-1^high^, *P* = 0.1085; severe versus mild cases, ICOS^high^, *P* = 0.1930). In other words, EV71 increased the frequency of circulating TFH cells with ICOS^high^ and PD-1^high^ expression that was not notably associated with the severity of patients in the acute stage of EV71 infection.

It is known that TFH cells are critical for promoting the development of antibody response by helper B cells in GC [10, 11]. Several seroepidemiologic investigations indicated that the neutralizing antibody can protect adults as well as children from EV71 infection once again, although the titers of specific neutralizing antibody were not correlated with severity of the disease with EV71 infection [3–6, 34, 35]. Why is that? To date, that is not well understood. Our data also indicated that the serum titers of specific NAb-EV71 in patients with acute stage of EV71 infection were higher than those in HC (severe or mild cases versus HC, *P* < 0.0001), but no significant difference was observed between mild and severe patients groups (severe versus mild cases, *P* = 0.9693), which corresponded to previous studies [3]. Additionally, we found that enhanced frequency of circulating CXCR5^+^CD4^+^ TFH, PD-1^high^CXCR5^+^CD4^+^ TFH, and ICOS^high^CXCR5^+^CD4^+^ TFH cells was notably positively correlated with the titres of specific NAb-EV71 in patients with EV71 infection, and a significant association was not observed between the mild and severe patients, respectively. These data also indicated that TFH cells with ICOS^high^ and PD-1^high^ expression participated in regulating antibody responses in the acute stage of EV71 infection, at the same time, which might give a good explanation to why there is no significant difference between the levels of specific NAb-EV71 in both mild and severe patients with EV71 infection.

Recent studies demonstrated that not only did TFH cells secrete IL-21, but IL-21 could also drive GC formation and TFH cell generation and function and promote terminal mature of B cell in GC, Ig class-switching, and antibody responses [10, 11, 20]. IL-6 has also induced IL-21 production and TFH cell generation, and mice lacking both IL-6 and IL-21 are unable to generate TFH cell-dependent immune responses [20, 21]. Bcl-6 transcription factor is selectively expressed in TFH cells, which is required for programming of TFH cell generation, and Bcl-6 expression is regulated by IL-6 and IL-21 [14, 21]. In current study, we found that the levels of sera IL-21 and IL-6 concentrations were significantly higher in both mild and severe patients with EV71 infection than those in HC (severe or mild cases versus HC, IL-21/IL-6, *P* < 0.0001), and they were found to be positively correlated with the frequency of circulating CXCR5^+^CD4^+^ TFH cells, respectively, which suggested that IL-21 and IL-6 might play an important role in the generation of TFH cells. In addition, a strongly positive correlation was observed between sera IL-21 and NAb-EV71 as well as between sera IL-21 and IL-6, indicating that IL-21 and IL-6 could promote the producing of specific NAb-EV71, and IL-6 might help in production of IL-21 cytokines. Furthermore, PCR analysis indicated that the expression of IL-21 and IL-6 mRNA was significantly increased in both mild and severe patients compared to HC (severe or mild cases versus HC, IL-21/IL-6 mRNA, *P* < 0.05). However, no difference of IL-21 and IL-6 cytokines and mRNA expression was found between mild and severe patients (severe versus mild cases, IL-21/IL-6 cytokines, *P* = 0.3909/0.3438; IL-21/IL-6 mRNA, *P* = 0.4887/08175); these findings indicated that the expression of IL-21 or IL-6 was not notably related with severity of disease with acute stage of EV71 infection, and the IL-6 high expression was consistent with pervious study [7]. Additionally, no significant difference of peripheral blood Bcl-6 mRNA expression among HC, mild, and severe patients is consistent with pervious study which might be associated with formation of memory of TFH cells [30, 37]. These data revealed that IL-21 and IL-6 participated in the generation of TFH cells and specific NAb-EV71 immune responses, which were not significantly associated with the severity of patients in the acute stage of EV71 infection.

In conclusion, our data indicated that increased frequency of circulating TFH cells with ICOS^high^ and PD-1^high^ expression was significantly and positively associated with serum specific NAb-EV71 level and IL-21 and IL-6 concentration in patients with EV71 infection, suggesting that TFH cells and associated cytokines might play a critical role in regulating humoral responses in the acute stage of EV71 infection. Further studies on the role of TFH cells in regulating B cell response and antibody production during the disease progression of EV71 infection will provide new insight into understanding the immune pathogenesis of EV71 infection in human.

## Figures and Tables

**Table 1:** Clinical characteristics of 60 recruited children with HFMD caused by EV71 infection.

Group	Number	M/F	Age (Months)	WBC (×10^9^)	CRP (mg/L)
Mild	29	16/13	46.41 ± 18.01	7.90 ± 1.84	6.95 ± 4.80
Severe	31	20/11	26.19 ± 12.98	13.11 ± 4.16	19.39 ± 11.91
HC	43	23/20	37.44 ± 11.14	6.34 ± 1.11	0.31 ± 0.10

Note: data correspond to the arithmetic mean ± SD. M/F: male/female; WBC: white blood cell; CRP: C-reactive protein; HC: healthy controls.

60 Laboratory-confirmed HFMD children with EV71 infection were recruited and classified into two groups according to the clinical severity of disease and degrees of neurological damage.
